# Ethical Reporting of Consent and Assent in Paediatric Oral Health Research in Malaysia: A Scoping Review

**DOI:** 10.21203/rs.3.rs-7565143/v1

**Published:** 2025-09-22

**Authors:** Tengku Nurfarhana Nadirah Tengku Hamzah, Nik Sherina Haidi Hanafi, Rumana Akhter Saifi

**Affiliations:** Department of Paediatric Dentistry and Orthodontic, Faculty of Dentistry, Universiti Malaya, 50603, Kuala Lumpur, Malaysia; Department of Primary Care Medicine, Faculty of Medicine, Universiti Malaya, 50603, Kuala Lumpur, Malaysia; Department of Social and Preventive Medicine, Faculty of Medicine, Universiti Malaya, 50603, Kuala Lumpur, Malaysia

**Keywords:** Paediatric Oral Health, Research Ethics, Ethical reporting, Child Assent, Parental Consent

## Abstract

**Background:**

Paediatric oral health research in Malaysia is governed by international framework alongside national requirements. Reporting of ethics approval and parental consent is well established, but child assent remains inconsistently documented. The frequent classification of dental studies as minimal risk may allow expedited review or consent waivers, raising concerns about transparency. This scoping review therefore aimed to map the reporting of ethics approval, parental consent and child assent in Malaysia paediatric oral health research published between 2001 and 2025, with a particular focus on describing current practices and documenting how assent procedures are reported in the absence of mandated requirements.

**Methods:**

This review was conducted and reported in accordance with the PRISMA Extension for Scoping Reviews (PRISMA-ScR): Checklist and Explanation guideline. An electronic search of five databases: Pubmed, Web of Science (WOS), SCOPUS, MyJurnal and the National Medical Research Registry (NMRR) was performed. Eligible studies included empirical research involving children aged 0–17 years in Malaysia. Data extraction focused on ethical approval, parental consent, and child assent. The transparency of assent reporting was assessed using a structured three-point framework informed by MREC guidelines for children aged 7–17 years.

**Results:**

A total of 72 articles met the inclusion criteria. Of these, 88.9% (n = 64) reported ethics committee approval and 93.1% (n = 67) documented parental consent, whereas child assent appeared in only 6.9% (n = 5). Reporting of ethics approval and parental consent increased substantially, rising from below 50% in 2001–2005 to above 95% after 2015. Child assent was not reported until 2021–2025, appearing in 17.9% of studies during this period. Of the five studies reporting assent, three used written forms, two relied on implied assent, and only one provided a detailed procedure aligned with ethical standards.

**Conclusions:**

Ethical committee approval and parental consent are now routinely reported in Malaysian paediatric oral health research, demonstrating broad compliance with international and national frameworks. However, documentation of child assent remains limited and often lacks procedural detail. Strengthening ethical transparency requires standardised, age-appropriate assent procedures and consistent editorial requirements. Improving reporting practices will enhance the protection of children’s developing autonomy, reinforce responsible conduct of research, and promote greater trust in paediatric oral health research.

## Background

Paediatric research plays a critical role in advancing clinical knowledge and improving healthcare outcomes for children. However, the inclusion of children in research associates with unique ethical obligations due to their inherent vulnerability and evolving cognitive capacities (1, 2). International ethical frameworks, such as the Declaration of Helsinki (DoH) and the guidelines issued by the Council for International Organizations of Medical Sciences (CIOMS), reinforce the necessity of obtaining informed consent from legal guardians on one hand, and on the other, emphasize securing age-appropriate assent from child participants. Both DOH and CIOMS, underscore the need for parental informed consent and child assent, tailored to children’s developmental stages and cognitive capacities to ensure ethical conduct of research (1–4).While consent in research is defined as informed and voluntary participation of an individual in a particular study; assent refers to a child’s voluntary and developmentally appropriate agreement to participate in a study, alongside parental or legal guardian consent.

Although paediatric medicine and paediatric oral health research are grounded in the same core ethical principles regarding consent and assent, distinct differences exist in their application and reporting practices. Paediatric medical research frequently involves clinical trials and invasive interventions that exceed minimal risk thresholds, necessitating rigorous Institutional Review Board (IRB) oversight, comprehensive ethical scrutiny, and detailed documentation of informed consent and assent procedures (3). In contrast, paediatric oral health research often includes preventive or observational studies conducted in community or school settings, commonly classified as minimal risk. This categorisation of minimal-risk may contribute to less detailed or omitted documentation of assent and consent procedures in research publications. The limited evidence on consent and assent practices in paediatric oral health research reveals substantial variability and inconsistencies in ethical reporting, which highlights the urgent need for greater transparency in upholding research integrity and ensure adequate protection for child participants (5).

Journals play a critical role in upholding ethical standards by mandating the explicit reporting of consent and assent procedures, thereby promoting transparency and enabling readers and regulatory bodies to verify adherence to established ethical norms (6–8). Transparent reporting of these ethical procedures is a cornerstone of responsible conduct of research (RCR), essential for maintaining integrity, accountability, and trust in research practices (2, 7). Clear and detailed ethical reporting enhances public confidence, protects the welfare of child participants, and ensures accountability among researchers and institutions (6, 7, 9). Quality reporting facilitates replication, critical appraisal, and systematic reviews, ultimately improving evidence-based practice in paediatric healthcare (2, 6). Structured guidelines such as the SPIRIT and CONSORT paediatric extensions (SPIRIT-C and CONSORT-C) further advocate clear documentation of child-specific ethical considerations (6).

Although the Malaysian Medical Research and Ethics Committee (MREC) mandates child assent for research involving minors, there is limited evidence regarding the consistency, quality, and level of detail in assent reporting. This gap is more apparent in Malaysian paediatric oral health research literature, highlighting a critical area for further investigation (16). The Academy of Medicine College of Paediatrics further underscores the need for flexible, culturally sensitive, and clearly articulated assent procedures that respect children’s agency within Malaysia’s diverse societal context (4). However, inconsistent enforcement of explicit reporting requirements by journals creates ambiguity regarding ethical compliance in paediatric research (10–12). While the absence of detailed consent or assent information does not necessarily indicate unethical practice, it does obscure transparency and weakens the rigor of ethical accountability (3, 8).

Transparent and high-quality ethical reporting in paediatric research, regardless of risk category, is not merely a procedural requirement but a fundamental safeguard for participant rights, ensuring methodological soundness, and fostering trust within the broader scientific and public communities (2, 6, 8). Identifying gaps in reporting can support researchers, ethics committees, policymakers and journal editors in strengthening ethical guidelines and publication practices, ultimately benefiting paediatric oral health research and broader healthcare practices (4). Addressing these gaps through clearer standards will be crucial for advancing ethically sound and methodologically robust paediatric research, thereby reinforcing both scientific integrity and ethical accountability (4). This scoping review aimed to systematically map the extent, nature, and transparency of reporting ethics approval, parental consent, and child assent in Malaysian paediatric oral health research published between 2001 and 2025, with a particular focus on describing current practices and documenting how assent procedures are reported. The guiding research question was: How are ethics approval, parental consent and child assent reported in Malaysian paediatric oral health research, and what patterns or gaps can be identified in the description of assent procedures?. Specifically, the review sought to describe the frequency of reporting of these ethical safeguards, to analyse reporting patterns across study settings and publication years and to explore the level of detail and transparency with which child assent procedures were documented.

## Methods

This scoping review adopted Arksey and O’Malley’s five-stage framework (15): (1) identifying the research question (2) identifying relevant studies, (3) selecting studies, (4) charting the data, and (5) collating, summarising, and reporting the results. The guiding research question for this scoping review was: “How are ethical safeguards including ethics approval, parental consent and child assent practices reported in Malaysian Paediatric Oral Health literature and to what extent do these reports transparently and ethically justify assent procedures?. Specific objectives include,(1) Quantify the frequency and proportion of Malaysian paediatric oral health research articles that report (i) ethical approval, (ii) parental consent, (iii) child assent (2) Identify the trends in ethical reporting classified by study settings and year of publication, grouped into 5 years interval (2001–2025) and (3) Assess gaps and transparency in child assent reporting through the application of a structured scoring framework. To achieve the third objective, a three-point scoring system was developed, guided primarily by the Malaysian Medical Research and Ethics Committee (MREC) requirements (16). The MREC guidelines specify assent requirements for children aged 7–17 years, which was taken into account in the assessment. A score of 0 was assigned when no mention of child assent was provided in the publication. A score of 1 was given when assent was mentioned but described vaguely or passively, for example implied through behaviour or briefly noted without specifying the format or justification. A score of 2 indicated clear and ethically adequate reporting, characterised by a description of the assent procedure that included the format (written or verbal), the age group of children involved, and explicit reference to alignment with ethical guidelines. For descriptive purposes, assent reporting was also categorised into written assent, verbal assent, passive or implied assent, assent not reported, and assent waived or deemed not applicable. This framework was intended to capture not only whether assent was reported, but also the quality and ethical adequacy of the reporting. This scoring system allowed for a systematic and transparent evaluation of the extent to which published studies documented assent procedures, distinguishing between studies that merely acknowledged assent and those that provided ethically sufficient detail. The reporting of this review adhered to the PRISMA Extension for Scoping Reviews (PRISMA -ScR) guidelines (14) to ensure methodological rigour. The review protocol and dataset are publicly accessible via the Open Science Framework (https://osf.io/gt96q.).

### Eligibility Criteria

Empirical studies (quantitative, qualitative, mixed methods) focusing on clinical, public health, or behavioural paediatric dental research involving participants aged 0–17 years, conducted in Malaysia and published in English or Bahasa Melayu between 01 January 2000 to 30 April 2025 were included. Non-empirical publications (editorials, opinions, reviews), adult only studies, animal/in vitro studies, secondary data analyses, and studies unavailable in full- text were excluded.

### Search Strategy

Five databases (PubMed, SCOPUS, Web of Science (WOS), MyJurnal, and the National Medical Research Registry) were systematically searched using Boolean operators (OR, AND) to combine terms related to population (children/adolescents) and concept (dental/oral health research). Detailed search strategies are provided in **Appendix 1**. The MyJournal and NMRR search was simplified to include “oral health” due to platform limitations. The final search was conducted on 9^th^ May 2025.

### Study Selection

**Figure 1:**
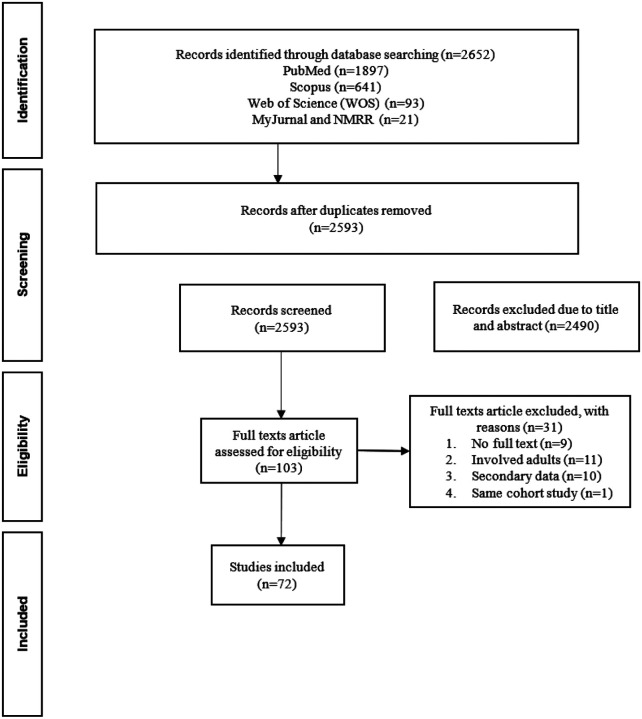
Selection process of the studies included based on PRISMA-ScR Flow Diagram

All retrieved citations were imported into EndNote X9 reference management software, where duplicates were identified and removed. The selection of the studies were conduted in two sequential phases (1) Initial screening of titles and abstracts and (2) Full-text screening for eligibility. The selection process is summarised in Figure 1, which outlines the number of records identified, screened, excluded, and included at each phase.

### Data Extraction

Data extraction was performed by a single reviewer (TNF) using a structured Excel form (**Appendix 2**). Two additional reviewers (NSH, RS) independently cross-checked data extraction accuracy, with discrepancies resolved through consensus. Extracted variables included study title, authors, publication year, journal type, Malaysian state, study design, participant age, and oral health focus. Variables associated with ethics included ethics approval, reporting of parental consent, and detailed child assent information such as format, justification for age thresholds, compliance with Malaysian MREC guidelines, and transparency score.

Ethical reporting in this review refers to the explicit documentation of ethics related practices within research publications, specifically including ethics approval, parental consent, and child assent procedures. The absence or inadequate reporting of these elements may reflect gaps not only in researchers’ practices but also in journal policies and editorial guidelines.

### Data Synthesis

Descriptive statistics were used to summarise the frequencies and percentages of studies reporting ethics approval, parental consent, and child assent. Data were analysed according to study design, participants age group (<7 years and ^3^7years), study settings and publication years in five-year intervals. Additionally, qualitative assessments were conducted to evaluate transparency scoring in studies that reported child assent, focusing on the clarity, comprehensiveness, and justification of the assent procedures.

## Results

**Figure 2:**
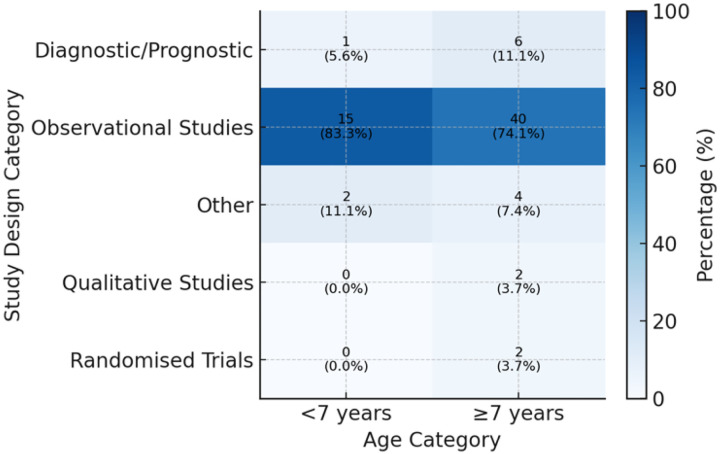
Distribution of study design by age category (<7 vs ^3^7 years) in Malaysian paediatric oral health research publications

Figure 2 displays the proportions of study design categories within two age groups. A total of 72 studies were included in this scoping review. Of these, 18 studies focused on children under seven years old, while 54 studies involved children aged seven years and above. Observational designs predominated in both age groups, accounting for 83.3% of studies in the under seven group and 74.1% in the seven and older group. Diagnostic and prognostic designs were less common, comprising 5.6% of the under-seven group and 11.1% of the older-child group. The “other” designs made up 11.1% and 7.4% of studies in the two age categories. Interactive methodologies such as qualitative interviews or participatory activities remain comparatively rare at 3.7% and are almost exclusively confined to studies of school aged children. Randomised trials appeared only in studies representing children aged seven years or older, comprising 3.7% of that group. These patterns suggest that while descriptive observational work underpins nearly all paediatric oral health research across all ages, more complex or interventional approaches are reserved for older school aged participants.

Table 1 summarises the frequency and percentage of studies reporting ethics committee approval, parental consent and child assent across eight research settings. Overall, 88.9% of studies (n=64) indicated that independent ethics committee approval had been obtained, and 93.1% (n=67) documented parental consent. By contrast, child assent was recorded in only 6.9% of studies (n=5). When stratified by study setting, research conducted in specialist clinic constituted the largest proportion (n=22;30.6%). All specialist clinic studies reported ethical approval and 95.5% (n=21; 29.2%) documented parental consent. However, only one study (1.4%) recorded child assent. Secondary schools setting accounted for 22.2% of the sample (n=16), with 93.8% (n=15; 20.8%) reporting ethical approval and 100% (n=16; 22.2%) reporting parental consent, yet only 4.2% (n=3) documented child assent. All preschool studies (n=7; 9.7%) uniformly reported both ethical approval and parental consent but did not report child assent. Primary school (n=12; 16.7%) and special care centre (n=9; 12.5%) studies similarly exhibited high rates of ethical approval (66.7%, n=8; and 88.9%, n=8, respectively) and parental consent (75%, n=9; and 88.9%, n=8), without any documentation of child assent being obtained. The single public dental clinic and the community programme studies, each reported parental consent, but neither reported child assent. However, the public dental clinic reported taking parental consent. Four studies (5.6%) classified as “Other” followed this overall pattern: three (4.2%) reported ethical approval, all four (5.6%) documented parental consent and only one (1.4%) recorded child assent. These results demonstrate that across all research settings, reporting of ethical committee approval and parental consent are the standard practice in Malaysian paediatric oral health research. However, the infrequent documentation of child assent even in studies involving older children, highlights a significant gap in current reporting practices.

**Figure 3:**
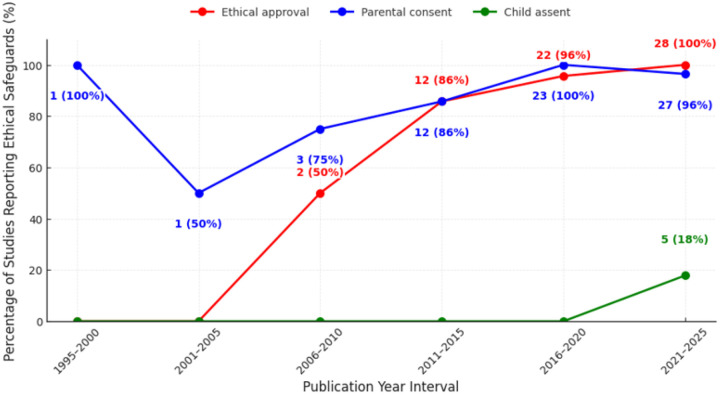
Trends in Reporting of Ethical approval, parental consent and child assent in Malaysian Paediatric Oral Health Publications by 5-year Intervals

Figure 3 shows the trajectory of studies reporting ethics approval, parental consent, and child assent across publication intervals beginning with the 1995–2000. Although the review timeframe was 2000–2025, one study published in 1997 was included as it fulfilled all other inclusion criteria; for consistency, it is presented within the earliest interval (1995–2000). In this study, only parental consent was reported, whereas ethics approval was not documented. The absence of child assent reporting is consistent with the regulatory context, as the Malaysian Medical Research and Ethics Committee (MREC) introduced specific requirements for assent in clinical research only in 2011. In the 2001–2005 interval, reporting of parental consent declined to 50%, and ethics approval remain unreported. In 2006 to 2010, half of studies (50%) reported ethical approval, and three quarters (75%) reported parental consent, signaling the influence of emerging research governance. Between 2011 and 2015, both ethical approval and parental consent were reported in 85.7% of studies, and these proportions climbed to 95.7% and 100% respectively in 2016 to 2020. Child assent was not mentioned in any studies until the most recent times. In 2021 to 2025, all studies reported ethical approval, 96.4% reported parental consent, but only 17.9% documented child assent. These trends show a steady improvement in the reporting of ethical approval and parental consent over three decades, with near universality achieved by 2020. In contrast, child assent remains rarely reported, appearing only in recent studies and at a low frequency. This highlights a persistent gap in meaningful engagement of children in research decision-making.

As shown in Table 2, a total of five Malaysian paediatric oral health studies explicitly reported obtaining child assent. Four of these studies (80%) were conducted in secondary schools or combined primary and secondary settings, and one study (20%) took place in a specialist clinic. All study participants were aged 9 to 17 years, in line with the Malaysian Medical Research and Ethics Committee guidelines. Three studies (60%) used written assent forms signed by participants, and two studies (40%) relied on implied assent inferred from participation. Only one study (20%) provided a clear, detailed description of the assent procedure and distinguished child assent from parental consent, earning the highest transparency score of 2. The remaining four studies (80%) mentioned obtaining assent without describing how it was obtained or differentiate it from parental consent, receiving a transparency score of 1. None of the studies justified the chosen age threshold for assent, although all followed the MREC age range. These results show that while child assent is beginning to be documented in Malaysian paediatric oral health research, detailed and transparent reporting of procedures and ethical justification for age thresholds remains limited.

## Discussion

### Summary of Key Findings

This scoping review examined ethical reporting practices across 72 Malaysian paediatric oral health publications. High compliance was observed for ethical approval (88.9%) and parental consent (93.1%), reflecting robust adherence to established ethical safeguards. In contrast, child assent was reported in only 6.9% of studies, and the level of transparency in describing assent procedures was limited. Among the few studies documenting child assent, only one (20%) provided a clear and comprehensive description of the assent process, achieving the highest transparency score. The remaining studies provided minimal procedural details, indicating a substantial gap in transparency and reporting of assent. Observational study designs predominated in both younger (< 7 years; 83.3%) and older (> 7years; 74.1%) age groups, with randomised trials and qualitative approaches comparatively rare and mostly involving older school-aged children. Variation was also noted by study setting: secondary school based studies documented of child assent more often (4.2%) compared to those conducted in specialist clinics (1.4%). No preschool or primary school based studies reported obtaining child assent, highlighting critical gaps in ethical reporting even though older children are capable of providing meaningful assent. These findings underscore a clear divergence between current reporting practice and international ethical guidance. Both the Declaration of Helsinki (DoH) and the Council for International Organizations of Medical Sciences (CIOMS) emphasise that child assent, alongside parental consent, is a core safeguard that respects children’s developing autonomy. Similarly, the Malaysian Medical Research and Ethics Committee (MREC) requires assent for participants aged 7–17 years, reflecting national alignment with these global principles. The limited and inconsistent documentation of assent observed in this review therefore highlighths an important gap in translating established ethical guidance into research reporting practices.

### Ethical Reporting and Responsible Conduct of Research (RCR)

The findings of this review highlight significant implications for ethical reporting within the framework of Responsible Conduct of Research (RCR). The low rates of explicit documentation and limited transparency concerning child assent practices in Malaysian paediatric oral health research suggest persistent gaps in ethical reporting. Good ethical reporting a cornerstone of RCR requires clear, explicit, and comprehensive documentation of research safeguards, particularly when involving vulnerable populations such as children. Transparent reporting is not only vital for the reproducibility and integrity of research but also for maintaining public trust in scientific inquiry. The lack of detailed reporting observed in this review may reflect limited awareness or understanding of assent requirements among researchers, or practical and cultural barriers that impede explicit documentation. Literature emphasizes the ethical importance of articulating assent procedures as a mean of recognising children’s developing autonomy and moral agency (22, 23). Transparent documentation allows assessment of the ethical quality of research practices and demonstrates respect for children’s evolving decision-making capacities.

Strengthening ethical reporting in line with RCR principles will require targeted educational training to improve researcher competencies in documenting assent, as well as institutional and editorial guidelines that encourage or require more detailed reporting. Addressing these gaps would ensure that ethical practices are not only undertaken but also demonstrably documented, thereby safeguarding the rights of child participants and reinforcing the integrity and accountability of paediatric oral health research.

### Alignment and Discrepancies with International and National Guidelines

Ethical safeguard reporting in Malaysian paediatric oral health research aligns closely with international standards, including the DOH and the CIOMS, as well as national ethical guidelines established by the Medical Research and Ethics Committee (MREC/NMRR) and the National Institutes of Health Malaysia (16). This alignment is reflected in the sustained, near-universal reporting of ethics approval (88.9%) and parental consent (93.1%) across the review period, suggesting effective implementation of these frameworks. Despite this strong overall alignment, significant discrepancies remain in the explicit documentation of child assent, an ethical safeguard highlighted in both international guidelines (DOH, CIOMS) and reinforced by national requirements (16). These international frameworks emphasis that child assent is not merely procedural but represents a fundamental acknowledgement of children’s developing autonomy. In Malaysia, explicit requirements for documenting child assent were further articulated through the Malaysian Code of Responsible Conduct in Research (MCRCR) endorsed by the National Science Council in 2017. However, adherence to these requirements appears inconsistent, reflecting potential gaps in awareness, understanding, or practical implementation among researchers (12).

The 2024 position statement from the Malaysian Academy of Medicine’s College of Paediatrics directly addresses these gaps, recommending a flexible minimum age for assent (from nine year old), advocating culturally tailored procedures, and encouraging the use of visual aids and multimedia tools to enhance comprehension (4). These recommendation mirror international best practices, which emphasise culturally sensitive communication strategies and meaningful engagement with children, particularly in diverse socio-cultural context such as Malaysia (11, 13). Nevertheless, the low documentation rate of assent (6.9%) and the limited procedural transparency observed in this review indicate that further efforts are required to achieve fuller alignment between national practice and international ethical expectations. Addressing these gaps through enhanced training, clearer editorial guidance, and structured institutional support for culturally appropriate assent processes would strengthen adherence to both international and national ethical standards, while ensuring stronger protection and recognition of child participants rights.

### Influence of publication venue

This review found that 80% of studies documenting child assent were published in international journals, including the only study that achieved the highest transparency score. This suggests that more stringent editorial policies and rigorous peer review standards in international journals may encourage greater ethical transparency and more detailed reporting of assent procedures. In contrast, local Malaysian journals demonstrated lower transparency in reporting child assent. This discrepancy may partly reflect differences in editorial requirements and the degree of alignment with internationally recognised principles of Responsible Conduct of Research (RCR). Although many Malaysian journals require adherence to core RCR elements such as ethics approval, informed consent, conflict of interest disclosures, authorship criteria (7), and structured peer review, explicit reference to Malaysia’s Code of Responsible Conduct in Research (MCRCR) remains uncommon. This limited emphasis may contribute to inconsistencies and under-reporting, particularly with respect to child assent alongside parental consent (12). Differences in study settings may also influence completeness of assent reporting, as researchers may face practical challenges in formalising assent procedures in certain contexts. However, since this review did not directly assess protocol-level practices, these explanations remain speculative and warrants further investigation.

### Recommendations and Future directions

Based on identified gaps, several strategies are proposed to strengthen ethical safeguard practices in Malaysian paediatric oral health research. In the short term, efforts should prioritise the standardisation of child assent procedures. Ethics Committees and Institutional Review Boards (IRBs) could provide clear, user-friendly guidelines and accessible documentation templates that incorporate culturally appropriate communication and community engagement strategies (11, 13). Journal editor, both local and international, should also encourage more detailed reporting of child assent processes or require clear justification for their omission. Medium term strategies, spanning one to three years, should concentrate on capacity building. The Ministry of Health (MOH) and professional bodies such as the Malaysian Dental Council could deliver targeted workshops on the ethical importance of child assent, practical documentation approaches, and culturally sensitive engagement. At the same time, universities and research institutions should operationalise consent and assent practices within undergraduate and postgraduate curricula, embedding these principle into teaching, clinical training and assessment to strengthen future practitioners competence in safeguarding children’s rights. Long term initiatives, over the next three years or more, may involve comprehensive revisions of national ethical guidelines. Regulatory authorities, including MREC/NMRR and NIH Malaysia, should explicitly define child assent requirements, including age appropriate formats, documentation standards, and guidance tailored to Malaysia’s cultural context (16). Academic and professional organisations should also promote qualitative research to explore the perspectives of children, parents and researchers, thereby informing the continuous refinement of national standards. Collectively, these measures can improve ethical transparency, enhance the protection of child participants, and strengthen the credibility and integrity of paediatric oral health research.

### Limitations

As a scoping review, this study relied exclusively on published reports. Ethical procedures, such as obtaining child assent, may have been undertaken but not explicitly documented in the included publications. The absence of journal requirements for detailed descriptions of assent processes may have contributed to under-reporting, although genuine omission due to limited awareness or understanding among researchers is also possible. Consequently, the findings reflect reporting practices rather than actual practice, limiting conclusions about the true extent of adherence to ethical standards for child assent. Nevertheless, the review provides valuable insights into current reporting patterns and highlights opportunities for strengthening ethical transparency in paediatric oral health research.

## Conclusion

This review highlights consistent reporting of ethics approval and parental consent but identifies significant limitations in the documentation of child assent. Enhancing transparency through clearer reporting standards, structured ethical training, community engagement, and culturally sensitive communication strategies will be important for supporting alignment with international guidance, strengthening respect for children’s developing autonomy, and reinforcing the credibility of paediatric oral health research.

## Supplementary Material

This is a list of supplementary files associated with this preprint. Click to download.

- Appendix1.pdf
- Appendix2.pdf

## Figures and Tables

**Table 1:** Reporting of Ethical Safeguards in Malaysian Paediatric Oral Health publications by study setting (n = 72)

Study setting	Ethical approval n (%)	Parental Consent n (%)	Child assent n (%)
Specialist clinic	22 (30.6)	21 (29.2)	1 (1.4)
Secondary school	15 (20.8)	16 (22.2)	3 (4.2)
Preschool	7 (9.7)	7 (9.7)	0 (0)
Primary school	8 (11.1)	9 (12.5)	0 (0)
Special care center	8 (11.1)	8 (11.1)	0 (0)
Public dental clinic	1 (1.4)	1 (1.4)	0 (0)
Community programme	0 (0)	1 (1.4)	0 (0)
Other settings	3 (4.2)	4 (5.6)	1 (1.4)
**Total, n (%)**	64 (88.9)	67 (93.1)	5 (6.9)

**Table 2:** Child assent reporting and transparency scores in studies published between 2021–2025 (n=5)

Study Title & Authors	Year	Journals	Study area	Study Design	Study setting	Age Range (Years)	Consent & Assent Reported	Assent Format	Assent Procedure Described	Age Justified	MREC Age Group Followed
**Impact of dental caries and pain on children’s oral health-related quality of life:** **A preliminary study**	2024	JUMMEC (Local)	Selangor	Cross-sectional	Others (primary & secondary schools)	9–16	Yes	Written	Not described – stated assent obtained	No	Yes
Ismail, N. R., Abu Bakar, N., Hasmun, N. N., & Tan, S. K.											
**The development and psychometric properties of Malay language ChiTd Oral Health Impact Profile—Short Form 19 (ML COHIP-SF 19)**	2025	Healthcare (International)	Selangor	Validation study	Specialist clinic	9–16	Yes	Written	Yes – assent obtained alongside consent, forms provided	No	Yes
Ismail, N. R., Tan, S. K., Abu Bakar, N., & Hasmun, N. N.											
**Inadequate toothbrushing practice and associated factors among inschool adolescents in Malaysia: Findings from GlobarSchool Health Survey (GSHS) 2017**	2025	PLOS ONE (International)	Nation wide	Cross-sectional	Secondary schools	13–17	Yes	Implied	Mentioned that students and parents signed consent; no mention of assent	No	Yes
Mohamad Anuar, M. F., Mohamed, N., Awaluddin, S. M., & Yacob, H.											
**Consumption of Carbonated Soft Drinks and Association with Health Behaviours and Mental Health among Adolescents in Malaysia: Findings from 2022 Adolescent Health Survey (AHS)**	2025	BMC Nutrition (International)	Nation wide	Cross-sectional survey	Secondary schools	13–17	Yes	Written	Students signed consent form; participation required dual consent	No	Yes
Mohd Zaki, N. A., Lai, W. K., Sallehuddin, S., Sahril, N., & Salleh, R.											
**Effect of Malocclusion Severity on Oral Health Related Quality of Life in Malay Adolescents**	2021	Health and Quality of Life Outcomes (International)	Penang	Cross-sectional	Secondary school	13–16	Yes	Implied	Mentioned that students and parents received assent and consent forms	No	Yes
Elyaskhil, M., Ahmad Shafai, N. A., & Mokhtar, N.											

## Data Availability

The datasets generated and analysed during the current study are not publicly available but are available from the corresponding author on reasonable request.

## Abbreviations

DOH: Declaration of Helsinki
CIOMS: Council for International Organizations of Medical Sciences
ICMJE: International Committee of Medical Journal Editors
MCRCR: Malaysian Code of Responsible Conduct in Research
MREC: Medical Research and Ethics Committee
NMRR: National Medical Research Register
IRBs: Institutional Review Boards
